# Simple Questionnaires to Improve Pooling Strategies for SARS-CoV-2 Laboratory Testing

**DOI:** 10.5334/aogh.3126

**Published:** 2020-11-18

**Authors:** Sophie Schneitler, Philipp Jung, Florian Bub, Farah Alhussein, Sophia Benthien, Fabian K. Berger, Barbara Berkó-Göttel, Janina Eisenbeis, Daphne Hahn, Alexander Halfmann, Katharina Last, Maximilian Linxweiler, Stefan Lohse, Cihan Papan, Thorsten Pfuhl, Jürgen Rissland, Sophie Roth, Uwe Schlotthauer, Jürg Utzinger, Sigrun Smola, Barbara C. Gärtner, Sören L. Becker

**Affiliations:** 1 Institute of Medical Microbiology and Hygiene, Saarland University, Homburg/Saar, DE; 1 Institute of Pneumology at the University of Cologne, Bethanien Hospital, Clinic for Pneumology and Allergology, Centre of Sleep Medicine and Respiratory Care, Solingen, DE; 1 Institute of Virology, Saarland University, Homburg/Saar, DE; 1 Department of Anesthesiology, Intensive Care and Pain Therapy, Saarland University, Homburg/Saar, DE; 1 Department of Otorhinolaryngology, Head and Neck Surgery, Saarland University, Homburg/Saar, DE; 1 Swiss Tropical and Public Health Institute, Basel, CH; 1 University of Basel, Basel, CH

## Abstract

**Background:**

Liberal PCR testing for severe acute respiratory syndrome coronavirus 2 (SARS-CoV-2) is key to contain the coronavirus disease 2019 (COVID-19) pandemic. Combined multi-sample testing in pools instead of single tests might enhance laboratory capacity and reduce costs, especially in low- and middle-income countries.

**Objective:**

The purpose of our study was to assess the value of a simple questionnaire to guide and further improve pooling strategies for SARS-CoV-2 laboratory testing.

**Methods:**

Pharyngeal swabs for SARS-CoV-2 testing were obtained from healthcare and police staff, hospital inpatients, and nursing home residents in the southwestern part of Germany. We designed a simple questionnaire, which included questions pertaining to a suggestive clinical symptomatology, recent travel history, and contact with confirmed cases to stratify an individual’s pre-test probability of having contracted COVID-19. The questionnaire was adapted repeatedly in face of the unfolding pandemic in response to the evolving epidemiology and observed clinical symptomatology. Based on the response patterns, samples were either tested individually or in multi-sample pools. We compared the pool positivity rate and the number of total PCR tests required to obtain individual results between this questionnaire-based pooling strategy and randomly assembled pools.

**Findings:**

Between March 11 and July 5, 2020, we processed 25,978 samples using random pooling (n = 6,012; 23.1%) or questionnaire-based pooling (n = 19,966; 76.9%). The overall prevalence of SARS-CoV-2 was 0.9% (n = 238). Pool positivity (14.6% vs. 1.2%) and individual SARS-CoV-2 prevalence (3.4% vs. 0.1%) were higher in the random pooling group than in the questionnaire group. The average number of PCR tests needed to obtain the individual result for one participant was 0.27 tests in the random pooling group, as compared to 0.09 in the questionnaire-based pooling group, leading to a laboratory capacity increase of 73% and 91%, respectively, as compared to single PCR testing.

**Conclusions:**

Strategies that combine pool testing with a questionnaire-based risk stratification can increase laboratory testing capacities for COVID-19 and might be important tools, particularly in resource-constrained settings.

## Introduction

Infection with the severe acute respiratory syndrome coronavirus 2 (SARS-CoV-2) may give rise to coronavirus disease 2019 (COVID-19) [1], which originated in the central part of the People’s Republic of China, in late 2019. The disease rapidly spread to all continents except Antarctica. Having been declared as a pandemic by the World Health Organization (WHO) on March 11, 2020, COVID-19 now heavily affects most countries in the world. As of November 16, 2020, more than 54 million SARS-CoV-2 cases have been confirmed and more than 1.3 million people have died of COVID-19 worldwide [2]. COVID-19 may lead to severe pneumonia, and many healthcare systems have been overwhelmed by the high demand of laboratory diagnostic capacity, inpatient treatment facilities, and personal protective equipment.

The diagnosis of COVID-19 requires detection of SARS-CoV-2 in respiratory specimens, utilising reverse transcription polymerase chain reaction (RT-PCR) assays [3]. In addition to individual case detection, laboratory testing plays a pivotal role for monitoring and containment strategies that entail testing, tracing, isolation, and quarantine. In many parts of the world, there is a need to scale up laboratory testing capacities for SARS-CoV-2, to enable examinations of large population groups, including healthcare staff and members of other critical sectors.

To overcome the limited availability of test kits, reagents, laboratory infrastructure, and trained staff, pooling of specimens has been suggested as a strategy to increase the number of tests performed at reduced costs [4]. In brief, pooling consists of multiple samples being combined and tested in a single PCR reaction. If a pool tests negative, individual samples are all considered negative, while only positive pools require testing of all single samples to unambiguously identify the positive one(s). Preliminary investigations pertaining to detection of SARS-CoV-2 suggest high diagnostic accuracy even when ≥ 20 samples were pooled into one single PCR [5,6,7]. However, samples suitable for pooling as well as pool sizes and the setting-specific prevalence of the disease under consideration need to be carefully assessed to avoid high numbers of positive pools. Here, we report the development, evolution, and validation of a simple questionnaire to guide and improve pooling strategies for SARS-CoV-2 testing in a University hospital setting in Germany.

## Materials and Methods

On March 11, 2020, our University hospital in the southwestern part of Germany set up SARS-CoV-2 testing facilities for hospital employees returning from, or sojourning in, risk areas according to the definition put forth by the Robert Koch Institute (RKI) at the beginning of the COVID-19 pandemic. Additionally, SARS-CoV-2 testing was also offered to all employees with symptoms of respiratory tract infection. Furthermore, samples originating from outbreak investigations initiated by the regional public health authorities (e.g. screening of staff, residents, and inpatients in regional nursing homes and hospitals) and screening samples from police staff and other regional public services were processed in the new testing facility and adjacent laboratories.

Combined nasopharyngeal and oropharyngeal swabs (eSwab, Copan; Brescia, Italy) were obtained from all individuals, and advice was offered pertaining to correct infection prevention measures. Samples were processed using RT-PCR (cobas^®^ SARS-CoV-2 6800, Roche Diagnostics GmbH; Mannheim, Germany; and RealStar^®^ SARS-CoV-2 RT-PCR Kit 1.0 RUO, altona Diagnostics GmbH; Hamburg, Germany) for SARS-CoV-2 on the day of sampling, using a previously validated diagnostic protocol for individual specimens and pool testing, which has been described in detail elsewhere [6]. When the SARS-CoV-2 testing facilities were set up in our hospital, all individuals who registered to get a swab taken were invited to fill in the questionnaire on a paper format. Subsequently, the information was transferred into an electronic database. In the questionnaire, we specifically asked for (i) symptoms compatible with COVID-19, based on a literature search; (ii) history of staying in risk areas in the preceding two weeks; and (iii) contact to individuals with a positive SARS-CoV-2 test during the preceding two weeks. Based on the response pattern and recommendations put forth by RKI, the corresponding swabs were immediately assigned to different racks and subsequently forwarded to the diagnostic laboratories for testing as either single tests or in pools of varying sizes.

As the pandemic evolved, several updated versions of the questionnaire were generated, in order to better reflect the current epidemiology (e.g. omission of previous travel to specific European areas outside Germany as risk factor) and incorporate new insights into the clinical symptomatology (e.g. anosmia). Experiences made by physicians and laboratory technicians during the practical conduct of patient sampling and pooling in the laboratory also influenced the revision of the questionnaires. Changes made were discussed and jointly agreed on by members of the hospital’s Center for Infectious Diseases, which comprises the Institute of Medical Microbiology and Hygiene as well as the Institute of Virology. The different questionnaire versions used between March 18 and July 5, 2020 are provided in the Appendix.

In brief, the following stratification was used whenever questionnaire data were available to provide some clinical information: first, individuals with typical symptoms compatible with COVID-19 (e.g. loss of taste and odour, or symptomatic contact persons to individuals tested positive for SARS-CoV-2) triggering a single test (no pool); second, individuals with an unspecific symptomatology not highly suggestive of SARS-CoV-2 infection (e.g. limb pain), triggering a pool of five samples; third, returning travellers from risk areas for SARS-CoV-2 and asymptomatic contact persons, triggering a pool of 10 samples; fourth and finally, regular screening of asymptomatic hospital staff in high-risk departments (e.g. bone marrow transplant unit), triggering a pool of 20–30 samples.

## Results

**Figure 1:**
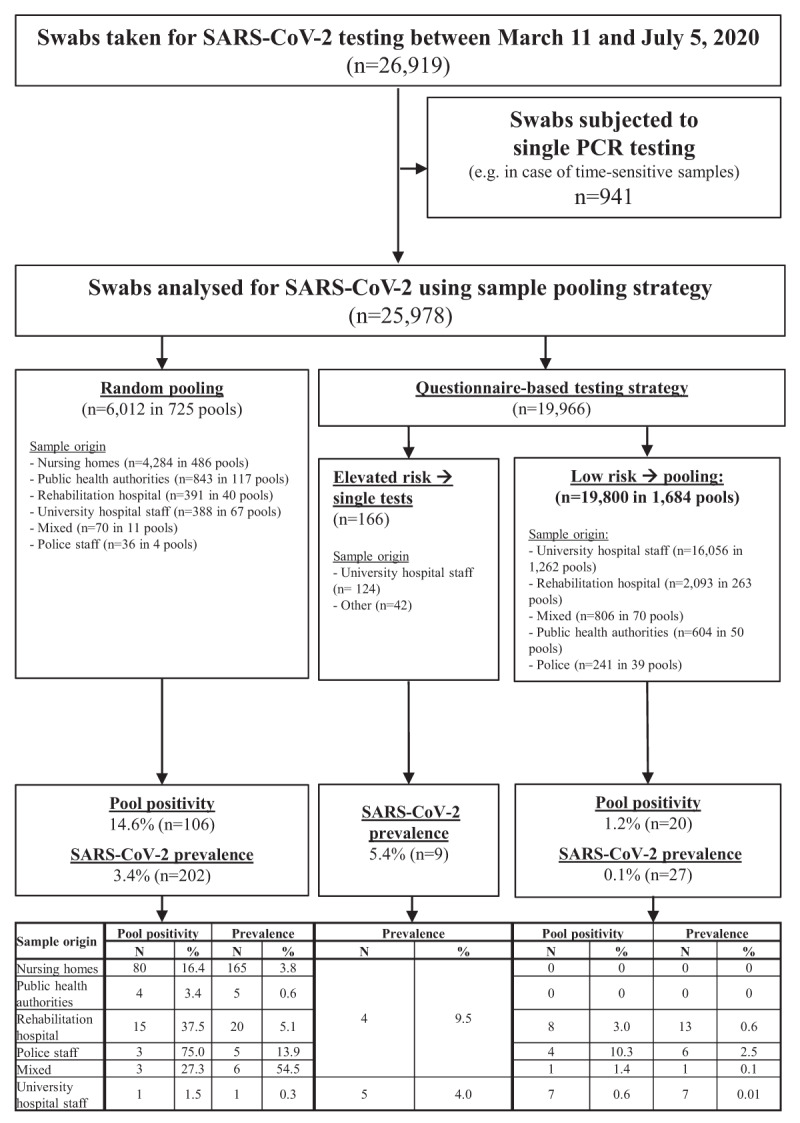
Sample flowchart for RT-PCR testing for SARS-CoV-2 using different sample pooling strategies at a University hospital in the southwestern part of Germany between March 11 and July 5, 2020.

Between March 11 and July 5, 2020, a total of 26,919 swabs were subjected to SARS-CoV-2 testing, of which 25,978 samples were processed using random pooling (n = 6,012; 23.1%) or questionnaire-based pooling (n = 19,966; 76.9%). Figure 1 shows the sample flowchart.

Overall, 238 individuals tested positive, owing to a SARS-CoV-2 prevalence of 0.9%. When comparing the different subgroups, the prevalence was highest (5.4%) in those individuals who had been questionnaire-stratified to single testing due to an elevated risk of COVID-19 according to their questionnaire response pattern (e.g. recent contact to a confirmed COVID-19 case or typical symptoms like anosmia). In the random pooling group, pool positivity and individual SARS-CoV-2 prevalence were 14.6% and 3.4%, respectively, whereas these were 1.2% and 0.1%, respectively, in the questionnaire group. When analysing the average number of PCRs needed to obtain the test result for one individual participant (derived from the sum of employed pool tests plus the number of subsequent individual PCR tests required to decode positive pools), this was 0.27 tests in the random pooling group, as compared to 0.09 in the questionnaire group (Table 1). The average number of individual test results produced by one PCR test was 3.8 in the random pooling group, as compared to 10.9 when questionnaire-based pooling was used.

**Table 1:** Comparative characteristics of (i) random sample pooling and (ii) a targeted pooling strategy based on a simple questionnaire for SARS-CoV-2 PCR testing in the southwestern part of Germany between March 11 and July 5, 2020.

	Samples tested in pools (n)	PCR pools (n)	Pool positivity rate (%)	Average number of PCRs needed to obtain an individual’s test result^1^	Average number of individual test results produced by one PCR test^1^

**Random pooling**	6,012	725	14.6	0.27	3.8
**Questionnaire-based pooling**	19,800	1,684	1.2	0.09	10.9

^1^ These calculated scores derive from the sum of employed pool tests plus the number of subsequent individual PCR tests required to decode positive pools.

We also performed a subgroup analysis of individuals with similar baseline characteristics from the same rehabilitation hospital during an outbreak with a relatively high SARS-CoV-2 prevalence (5.5%). Individuals were sampled over consecutive days, either using a random pooling or a questionnaire-based pooling approach. In the questionnaire group, the combination of initial pool tests and subsequent single PCRs required to decode the positive pools resulted in a lower quantity of actual PCRs (i.e. an average number of 35.6 total PCRs were required to assign a final test result to 100 individuals), as compared to the random pooling group (average of 43.3 PCRs needed to generate test result for 100 individuals; Table 2). The actual SARS-CoV-2 prevalence in individuals stratified by the questionnaire as low, moderate, and high risk of having acquired COVID-19 were 0%, 8.9%, and 27.6%, respectively.

**Table 2:** Comparative evaluation of questionnaire-based vs. random pooling in samples originating from the same rehabilitation hospital on consecutive days in March 2020 in the southwestern part of Germany.

Questionnaire used?	Assessed as	Pool size	Number of individuals	Number of pools	Pool positivity		Prevalence		PCRs needed to obtain 100 individual results

					N	%	N	%	

**Yes**	Low risk	10–13	103	10	0	0	0	0	
	Intermediate Moderate risk	5	45	9	3	33	4	8.9	
	High risk	Single test	29	0	–	–	8	27.6	
	Total		177				12	6.8	35.6
**No**		10	150	15	5	33	6	4.0	43.3

The following risk stratification procedure was used for patients and staff of the rehabilitation hospital: *Low risk*: **Neither** clinical symptoms **nor** known exposure to an individual with a positive SARS-CoV-2 test result *Intermediate Moderate risk*: **Either** clinical symptoms **or** known exposure to an individual with a positive SARS-CoV-2 test result *High risk*: New-onset clinical symptoms **and** known exposure to an individual with a positive SARS-CoV-2 test result

## Discussion

Our experience underscores the potential of pooled multi-sample diagnostics to enhance laboratory testing for SARS-CoV-2. Additionally, we found that a simple questionnaire, which requires less than three minutes to be filled in, facilitates stratification of samples with different pre-test probabilities of being positive in separate pools. Furthermore, samples of individuals with an elevated probability of testing positive for SARS-CoV-2 could be directly subjected to single testing, which has the potential to reduce the time to result and decrease the number of positive pools and thus the required additional single PCR tests to decode these pools.

Our findings have important ramifications for microbiological laboratories that are confronted with a high amount of SARS-CoV-2 testing requests. A recent survey elucidated considerable differences with regard to laboratory preparedness for COVID-19 across Europe [8], which may lead to significant underestimations of the true SARS-CoV-2 infection rates [9]. In addition to individual patients, COVID-19 testing is now also used to screen healthcare workers, returning travellers, and public service officials [10], thus exacerbating the need for additional diagnostics. Shortage of reagents has already led to alternative workflows in some countries [11]. Hence, validated pooling strategies should be employed more widely, particularly in laboratories that are overwhelmed by high requests for testing and in resource-constrained settings [12]. Indeed, a recent analysis pertaining to low-resource areas concluded that pooled group testing is more efficient than individual testing and “at a time when tests are in short supply globally, is a more socially responsible strategy” [13]. Even in high-income countries, the rapid identification of individuals at highest risk and targeted individual testing outside pools may accelerate the time to a conclusive test result, thus overcoming the potential disadvantage of pools that may arise from the delayed time to identification of the positive individual in one positive pool.

Effective pooling procedures are influenced by the prevalence of the disease under investigation and can significantly reduce the number of tests, the laboratory workload, and associated costs [14]. Considering the low prevalence of SARS-CoV-2 in our study (0.9%), laboratory capacity might be further enhanced if pools containing more than ~5–10 samples are used. Indeed, the PCR protocol employed in our study was previously validated for detection of positive specimens in pools of up to 30 samples with high diagnostic accuracy [6].

Our study has several limitations. First, it is important to note that our samples stem from a heterogeneous study population (i.e. employees with risk factors and symptoms, screening of public service professionals, and nursing home-associated outbreak investigations). Hence, our results might not be representative for the general population. Second, as pool positivity and other parameters are influenced by the different prevalence of infection, a direct comparison between the two pooling strategies is difficult. Indeed, random pooling was mainly employed during the first weeks of SARS-CoV-2 testing in the study area (March 2020), when the prevalence was relatively high, whereas questionnaire-based pooling was set up in late March 2020 and was maintained throughout the study, when infection rates dropped considerably following broad lockdown strategies employed in Germany and neighbouring countries. Third, as the COVID-19 pandemic now affects most countries worldwide, the use of questions pertaining to recent history of staying in risk areas might not be appropriate anymore, and hence, could be omitted. Indeed, in response to the rapidly changing epidemiology of the pandemic, we constantly adapted and updated the questionnaire (see Appendix). Additionally, one might argue that it may not be necessary to document the presence of symptoms suggestive of COVID-19, because screening of asymptomatic persons is now also recommended in many countries. However, individuals with typical symptoms are likely to have a higher pre-test probability of a positive SARS-CoV-2 test, and could thus be directly tested as individual swabs instead of being allocated to a pool of samples.

In conclusion, strategies that combine pool testing with a questionnaire-based risk stratification can increase laboratory testing capacities for SARS-CoV-2, and might be particularly useful in resource-constrained settings that are increasingly affected by the COVID-19 pandemic.

## Appendix

Different versions of a questionnaire to guide sample pooling strategies for SARS-CoV-2 PCR testing at a University hospital in the southwestern part of Germany between March 11 and July 5, 2020.
